# Dynamic-Distance-Based Thresholding for UAV-Based Face Verification Algorithms

**DOI:** 10.3390/s23249909

**Published:** 2023-12-18

**Authors:** Julio Diez-Tomillo, Jose Maria Alcaraz-Calero, Qi Wang

**Affiliations:** School of Computing, Engineering and Physical Sciences (CEPS), University of the West of Scotland (UWS), Paisley PA1 2BE, UK; julio.diez-tomillo@uws.ac.uk (J.D.-T.); qi.wang@uws.ac.uk (Q.W.)

**Keywords:** face verification, thresholds, siamese network, cosine distance, Euclidean distance

## Abstract

Face verification, crucial for identity authentication and access control in our digital society, faces significant challenges when comparing images taken in diverse environments, which vary in terms of distance, angle, and lighting conditions. These disparities often lead to decreased accuracy due to significant resolution changes. This paper introduces an adaptive face verification solution tailored for diverse conditions, particularly focusing on Unmanned Aerial Vehicle (UAV)-based public safety applications. Our approach features an innovative adaptive verification threshold algorithm and an optimised operation pipeline, specifically designed to accommodate varying distances between the UAV and the human subject. The proposed solution is implemented based on a UAV platform and empirically compared with several state-of-the-art solutions. Empirical results have shown that an improvement of 15% in accuracy can be achieved.

## 1. Introduction

Nowadays, face recognition and face verification are widely deployed in many areas of our lifes, for instance, at border control points between countries, for building access management in universities and companies, unlocking smartphones, service authentication/authorisation for individuals, and so on. Face recognition aims to identify all the concerned persons, and thus a database is usually needed to store all the people to be identified. In contrast, face verification seeks to identify only one person, so a database would not be needed as only one person would be being searched. The reasons for recognising or verifying a face are many, including customised services, targeted advertisement, health measures, or even security.

Moreover, unmanned aerial vehicles (UAVs) are increasingly deployed in multiple areas for Search and Rescue (SAR) operations [1], delivering goods [2], and many other purposes, as a cost-effective mobile platform. When UAVs meet face verification, the possibilities of potential use cases are immense in light of joining the capabilities of both technologies together in one platform. It is therefore promising to combine the two technologies and be able to recognise people from UAVs. Nevertheless, it is important to secure the UAV, as it will process sensitive privacy data, like the faces images for verification purposes [3].

Such a face verification-enabled UAV system allows for the recognition of a person from distances, without being intrusive to the user by removing the need for a camera close to the face, and opens up opportunities for new location or mobility-based services. Combining both technologies, face verification and UAVs could be highly useful for many use cases, lowering costs and extending the range. One main new use case for face verification from UAVs is the Drone Guard Angel for public safety escort services in the Arcadian-IoT European Project [4]. In this use case, a UAV comes to the customer’s position on demand, recognises the person, verifies his/her face to authorise the services as subscribed, and accompanies the person home in a safe way, acting as an invigilator that can help in case of any malicious activity.

Currently, most face verification techniques are performed at close distances. For instance, when a phone is unlocked using face verification, it is usually positioned 30 cm from the face. At this distance, all the features of the face can be captured with a high resolution; therefore, face verification can be performed without significant difficulties using state-of-the-art technologies. However, if face verification is performed from a UAV, it is not possible to position it at 30 cm from the face due to safety reasons. Thus, the UAV has to be at long distances, which vary depending on its size. It is noted that most face verification algorithms are optimised for close distances. Moreover, face verification is usually effective at a fixed distance or within a small range, whereas, for a UAV-based use case, the distance between a face and the UAV is significantly higher; thus, the size of the face in pixels varies and the low pixel resolution of a face introduces significant difficulties in face verification, imposing the challenge of developing new techniques to improve the accuracy of these algorithms when face verification is carried out from longer distances.

In addition, for services like the Drone Guard Angel, the operational environments pose further challenges in terms of poor lighting conditions due to outdoor and late evening operations as well as the fast-changing positions and angles of the target face due to the movement of the UAV. For this reason, a new technique that is lighting- and position-adaptive is proposed in this manuscript to enable the successful deployment of the new services such as Drone Guard Angel.

Consequently, the main contribution of this work is a novel dynamic size-adaptive threshold technique for face verification from UAVs, where the face has to be verified from long distances and in a highly adaptive manner in response to the challenging operational environments where lighting conditions and the positioning of the face change rapidly. To this end, a smart pipeline has been developed for introducing dynamic thresholds.

Furthermore, in order to compare the proposed technique with the existing solution, an empirical analysis of different algorithms is performed, including four state-of-the-art face verification algorithms (ArcFace [5], VGG-Face [6], SFace [7], and Dlib [8]). To conduct a more complete analysis, two different similarity metrics for distance calculation are explored: cosine and Euclidean distances. A study of the suitability of each metric for the different face verification algorithms is conducted too.

The main contributions herein can then be summarised as follows:

- A novel adaptive threshold algorithm for enhancing different state-of-the-art face verification and recognition algorithms to suit UAV-based applications.
- A new pipeline with novel capabilities to integrate the proposed dynamic threshold algorithm.
- An empirical evaluation of the effectiveness of the proposed adaptive threshold technique when dealing with UAV-based face verification use cases using two different similarity metrics.

The rest of the paper is structured as follows: in Section 2, the literature in this research area is reviewed, especially the representative state-of-the-art face verification algorithms and metrics focused for enhancement in this paper. Section 3 describes the proposed design of the face verification pipeline. In Section 4, the algorithm to calculate the proposed thresholds, together with the dataset used, is explained. In Section 5, the implementation of the experiments is presented. In Section 6, the accuracy of the algorithms of the proposed dynamic-distance-based thresholds is compared with the state-of-the-art algorithms and the suitability of each metric for the algorithms is analysed. Finally, in Section 7 conclusions are drawn.

## 2. Related Work

Most of the state-of-the-art studies focus on face recognition rather than on face verification, whilst the latter is what this paper will emphasise. The primary distinction between them lies in the outcome: a face recognition algorithm provides the identity of the recognised person, whereas a face verification algorithm determines whether the person being assessed matches the intended individual or not.

Furthermore, the inference time of face recognition algorithms is always greater than that of face verification ones. This is due to the need to compare one face with all the present faces in the database to achieve face recognition. On the contrary, in face verification it is only necessary to compare one face to another to decide if they are or are not the same person.

Additionally, the decision could be made differently between face recognition and face verification. For the former, the decision can be performed through a K Nearest Neighbour (KNN) algorithm. It stores all the available classes and classifies the data based on similarity. The decision it makes relates to which person in the database is most similar to the one being compared to. On the other hand, face verification is based on a global threshold to decide if it is the same person or not [9]. For this reason, choosing an optimal threshold is critical. If it is not defined properly, many false positives or negatives are possible, leading to erroneous verification. Other thresholding techniques are based on having client-specific thresholds. This paper is based on applying different thresholds depending on the person to be identified [10].

**Figure 1:**
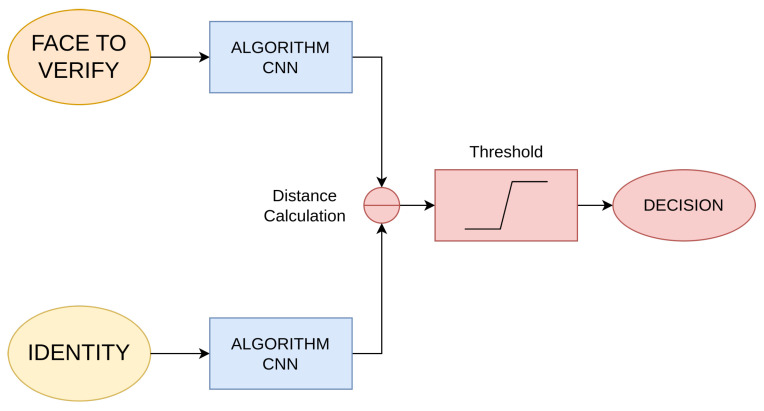
Simplified diagram of the face verification process.

Figure 1 shows the most common approach used by other research works to verify a person: a Siamese neural network [11]. In other words, two identical neural networks with the same network model/structure and the same weights. Two images are also needed. The first one is a picture of the face of the person already verified. The second one is a picture of the person to be verified. These pictures go through the Siamese neural network, obtaining as output two different vectors that can be compared as they are the result of the same neural network. Then, the distance between them is calculated. If the distance is above the threshold, the decision is that they are not the same person. If it is below the threshold, it is concluded that they are the same person.

Currently, there is a lack of literature on face verification from UAVs. A few studies can be found about detecting and finding people from UAVs [12], for instance, to conduct SAR operations [1]. There are studies in which face verification from UAVs is performed, although only from very short distances. Moreover, they focused on traditional face verification algorithms with low accuracy but high speed, such as LBP (Local Binary Pattern) [13]. Other papers develop algorithms for face verification; however, they do not use real images from UAVs.

For example, [14] uses a Haar Cascade for detecting faces, and an LBPH (LBP Histogram) Face Recognizer. A complete system for the acquisition of images from a UAV and their subsequent physical recognition is proposed. The results do not provide any real images from the UAV, so it has not been possible to test it in a real system. While the LBPH algorithm is easy to implement, it is not as accurate as more current face verification algorithms such as those selected in our contribution.

Another similar work is [15], which also employed an LBPH algorithm along with OpenCV to perform face recognition from a UAV. It used a Raspberry Pi and a Raspberry Pi Camera. The results present some images of face recognition while controlling the UAV. Although all the images are from very close distances, we are trying to perform face recognition from long distances, up to 30 m.

Several studies have explored both the influence of the distance from the UAV to the person and the angle between them. Certain findings suggest that the distance and the depressing angles are crucial constraints in face verification systems, particularly when the UAV is positioned at high altitudes [16]. Another study analysed the effects of the horizontal and vertical angles between the face and the camera, utilising a face recognition pipeline based on OpenCV and Dlib libraries [17]. Further research has developed an innovative deep learning-based face detection and recognition system for drones. The system was analysed by varying the distance and height of the drone to the person, demonstrating good accuracy at lower heights and close distances [18]. These existing studies did not address the adaptive dynamic thresholding technique proposed in this paper.

### 2.1. Empirical Comparison of Face Verification Algorithms

Table 1 shows an in-depth comparison among different face verification and recognition algorithms available in the literature. The verification performance and the different parameters of each algorithm have been obtained from its official paper. The table compares the input and output size of the network used by each algorithm, and the verification performance using two different datasets: Labeled Faces in the Wild (LFW) [19] and YouTube Faces (YTF) [20], as well as comparing the size of the pre-trained weights of the neural networks, and the number of images it has been trained with.

The human-level performance in face verification on LFW is 97.53% [21]. Four algorithms shown in the table do not surpass this value: Open-Face [22], Deep-Face [23], Fisher-Vector Faces [24], and TL Joint Bayesian [25].

VGG-Face [6], Deep-Face [23], and CosFace [26] are the algorithms with the heaviest weights, leading to slow performance when executed. On the other hand, DeepID2 [27] and Open-Face [22] have the lightest weights. Therefore, they will be fast but not as accurate as others. Furthermore, TL Joint Bayesian [25] and GaussianFace [28] algorithms do not have pre-trained weights available, and they have not been evaluated on the YTF dataset [20].

AdaFace [29], CurricularFace [30] and SFace [7] are new algorithms that achieve good accuracy in the LFW dataset (more than 99%). However, there is no information about the output size or the accuracy on the YTF dataset for the first two.

Four representative algorithms have then been selected to perform deeper, empirical analysis in this paper, including VGG-Face [6], ArcFace [5], SFace [7], and Dlib [8].

These four algorithms have been selected for several reasons. Firstly, they all achieve more than 98% accuracy in the LFW dataset; therefore, all four surpass the human-level performance of LFW (97.53%). Moreover, they have pre-trained weights available that can perform tests without training. ArcFace was selected because it provides the loss function with the best verification performance of the three algorithms compared (SphereFace [31], CosFace [26], and ArcFace [5]), whilst Face-Net [9] has been discarded because SFace [27], Dlib [8], and VGG-Face [6] have better verification performance. Furthermore, SFace explores a new method by training the model with synthetically generated data.

These four selected algorithms have the greatest potential to improve through training as they all have open-source code that can be modified according to our needs, and the datasets used are public so the results of these four algorithms can be replicated and used conveniently.

These face verification models are Convolutional Neural Networks (CNNs). They obtain an image as an input and have a vector as an output. They represent images of faces as vectors. The images captured with the UAV have to be resized in order to have the same size as the inputs for each algorithm. Then as output shapes, there is a vector with different sizes for each algorithm. These vectors will be the ones used to measure the distance between them to decide if the person is verified or not. The bigger the output shape, the greater the computational usage and processing time but the higher the accuracy.

**Table 1:** Comparison between different face verification state-of-the-art algorithms.

Algorithm	Input Size	Output Size	Verification	Performance	Pre-Trained	Training	Other Information
			**on LFW Dataset**	**on YTF Dataset**			
**Human-beings** [21]	N/A	N/A	97.53	NG	N/A	N/A	Humans performance
**Open-Face** [22]	96 × 96 × 3	128	92.92	NG	14 MB	1 M	Open-source framework
**Deep-Face** [23]	152 × 152 × 3	4096	97.35	91.40	551 MB	4.4 M	Deep Convolutional Network
**Fisher-Vector Faces** [24]	160 × 125 × 3	128	93.03	NG	NG	NG	Fisher Vectors
**TL Joint Bayesian** [25]	Variable	NG	96.33	NG	NG	NG	Bayesian model
**VGG-Face** [6]	224 × 224 × 3	2622	98.95	97.30	554 MB	2.6 M	Convolutional Neural Network
**CosFace** [26]	112 × 96 × 3	NG	99.73	97.60	214.3 MB	5 M	Loss function
**DeepID2** [27]	55 × 47 × 3	160	99.53	93.20	1.6 MB	0.2 M	Deep Neural Network
**GaussianFace** [28]	150 × 120 × 3	NG	98.52	NG	NG	NG	Gaussian Model
**ArcFace** [5]	112 × 112 × 3	512	99.83	98.02	131 MB	5.8 M	Loss function
**Dlib** [8]	150 × 150 × 3	128	99.38	NG	22 MB	3 M	Machine Learning Toolkit
**SphereFace** [31]	112 × 96 × 3	512	99.42	95.00	68.6 MB	0.5 M	Loss function
**Face-Net** [9]	160 × 160 × 3	128	98.87	95.12	88 MB	200 M	Deep Convolutional Network
**AdaFace** [29]	112 × 112 × 3	NG	99.82	NG	668 MB	15.1 M	Loss function
**SFace** [7]	112 × 112 × 3	128	99.13	NG	36.9 MB	1.1 M	Synthetic dataset training
**CurricularFace** [30]	112 × 112 × 3	NG	99.80	NG	249 MB	6.3 M	Loss function

NG = not given; N/A = not applicable.

#### 2.1.1. VGG-Face

This algorithm has been developed by the University of Oxford. Its architecture has 22 layers and 37 deep units. Also, the input is a face image of 224 × 224 pixels, which is the biggest input of the four algorithms analysed. Moreover, it also has the biggest output, a 2622 dimension vector. That is why, as will be seen below, this algorithm achieves the best accuracy but it is also the slowest [6].

#### 2.1.2. SFace

It has been trained using a synthetically generated face dataset to train the face verification model. To generate the data, a class-conditional generative adversarial network has been used. The generated dataset is composed of 634k synthetic images distributed on 10,575 classes. The face verification model uses ResNet-50 as the backbone and the loss function used is CosFace. The input size of the face is 112 × 112 pixels  [7].

#### 2.1.3. ArcFace

It is an algorithm developed by the Imperial College London. It is based on MXNet [32] and Python, but in the framework used it is based on a re-implementation in Keras [33]. The model is based on ResNet34 Architecture. The algorithm has as input an image of 112 × 112 pixels and has as output a vector of 512 dimensions [5].

#### 2.1.4. Dlib

It is not an algorithm but a library written in C++ that also has a Python interface. This library contains machine learning algorithms like the one used for face recognition. It is based on a Resnet34 model but with some modifications consisting of 29 convolutional layers. Its input is a 150 × 150 face image and its output is a 128 dimensions vector [8].

### 2.2. Distance Calculation Methods

For the calculation of the distance between the two images (the one of the person to compare and the image to be compared), two different metrics will be used: Euclidean distance and cosine distance. For each one of them, a different dynamic threshold will be calculated.

## 3. Proposed Pipeline Architecture of Face Verification Algorithm

**Figure 2:**
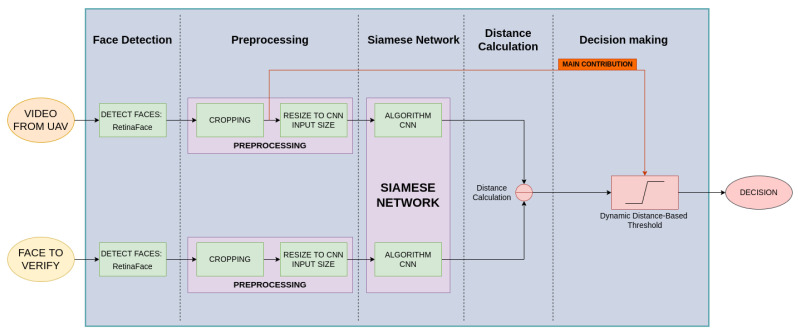
Block diagram of the proposed face verification pipeline composed of five stages: face detection, preprocessing, Siamese network, distance calculation, and decision making. There are two inputs: a video from a UAV and the face of the person to identify, and an output that is the decision.

This research develops a new system with a size-adaptive dynamic threshold for enhancing face verification capabilities and then compares four state-of-the-art face verification algorithms. Figure 2 shows the proposed face verification system. The proposed system needs two inputs to perform face verification. The first one is a video received from a UAV, and the second one is the face of the person to verify. Each input will go through the same steps in parallel up to the distance calculation. The system could be divided into five different stages as shown in the figure:

1. **Face Detection:** This is the first stage in our pipeline. The images are processed with RetinaFace [34], which is a face detection algorithm with high performance for both high- and low-resolution faces. Notice that face detection has nothing to do with face identification or face verification. Face detection is the process of determining that there is a face in a set of pixels. Thus, the output of this algorithm is the number of faces in the image and the coordinates of each of them in the image. For research purposes, there will be only one face in each frame. This stage is the slowest of the pipeline as the detection lasts on average 170 ms, causing the maximum processing speed to be less than 6 fps (frames per second). This face-detection algorithm has been selected for our pipeline because of its considerable accuracy in detecting low-resolution faces.
2. **Preprocessing:** It is divided into two different components. First, the detected face is cropped from the frame using the coordinates provided by RetinaFace and the OpenCV library. Then, the cropped face is resized to the input size of the algorithm that is going to be used according to Table 1 using OpenCV. This step is really fast as it only takes 0.3 ms to complete. In most cases, the image ratio of the cropped face will not be the same as the expected size of the algorithms. Therefore, in order not to distort the image, black pixels are included to reach the expected size while maintaining the same image ratio. Further preprocessing is not executed in order to compare the performance of the face verification algorithms at different distances without any image enhancement methods.
3. **Siamese network:** This stage leverages a Siamese network, which contains two CNNs. They have the same architecture and the same weights. Therefore, for example, if the same two images are introduced in the Siamese network the same outputs would be obtained in both. It can be used with any face verification algorithm. In this research, the four selected ones are explored. The inputs are the two cropped faces that are going to be compared with each other. The size of both images is the same as the input required for the CNNs. The outputs are the features of each face converted into a vector. The length of each vector is different according to the definition of the algorithm, as can be seen in Table 1.
4. **Distance calculation:** In this stage of the pipeline, the distance between the features of the faces is calculated. Two different metrics are used for the distance calculation: Euclidean distance and cosine distance, as described in the previous section. Meanwhile, more metrics can be explored, for instance, Manhattan distance. The metric used depends on the face verification algorithm in the Siamese network. Depending on how it obtains the features, one metric can perform better than others in determining if the faces are the same or not. Therefore, each algorithm has a better performance with one of the metrics.
5. **Decision making:** This is the most critical stage where a decision is made based on a threshold. If the distance is above the threshold, the faces are from different people; otherwise, they are the same person. Thus, it is crucial to define an appropriate threshold to be able to have the true positives while minimizing the false negatives. Our main contribution at this stage is a size-adaptive dynamic threshold; depending on the size of the cropped face from the video, different thresholds are used. Just as the size of the image varies, so do the features obtained from the Siamese network. Thus, the distances between the faces will vary too. That is why a dynamic-distance-based threshold will improve the accuracy of the face verification algorithm, leading to more true positives and fewer false negatives. A different threshold will be used depending on the selection of the algorithm, the metric, and the distance between the face and the UAV (calculated in the last stage). Finally, the pipeline ends by showing the decision that is made: whether it is the same person or not. More details of the proposed threshold algorithm are presented in the next section.

## 4. Proposed Dynamic-Distance-Based Thresholding

The problems of the existing face verification algorithms and the distances at which they are least efficient have been identified and can be improved by defining appropriate thresholds for each distance.

In this section, a scale of sizes is defined and then the proposed distance-based thresholds are calculated for each range using a new algorithm. The calculations are data-driven as the thresholds are calculated based on the data obtained from a dataset.

### 4.1. Dataset Description

A new dataset of UAV-recorded human subjects has been created. This is due to the need for a dataset with a list of specific characteristics for our research and use case, and the fact of the lack of public availability of such an existing dataset.

This new dataset is composed of videos recorded from a UAV at different distances. The UAV used for the recording is a DJI Mini 2. The videos obtained have a resolution of 4K (3840 × 2160 pixels) and a frame rate of 30 fps.

**Figure 3:**
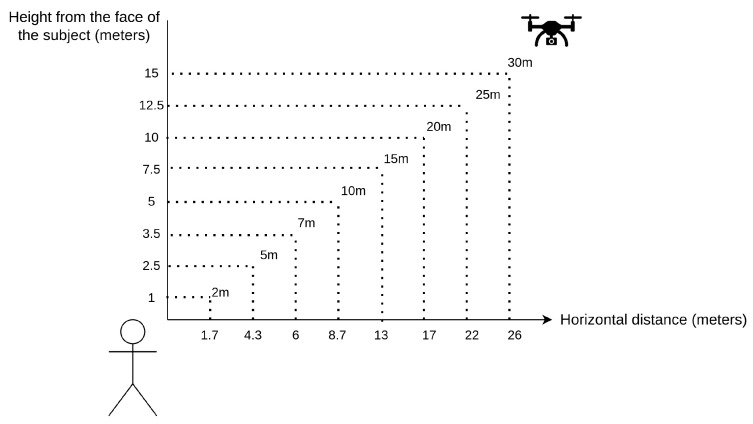
Schematic of the dataset recording distances.

For the purpose of this research, 20 volunteers were recorded individually in an open field with no one else in the video—one face per video. Each video lasted 30 s, during which a human subject was asked to make head movements to obtain different angles of the face and to stare at the camera for a few seconds. Per subject, eight videos were recorded with each subject from different distances (2, 5, 7, 10, 15, 20, 25, and 30 m). Figure 3 shows the distances arranged for the recordings. The distances are measured from the face of the subject to the UAV camera, and in all cases the UAV has 30 degrees of elevation above the face of the person. The dataset contains 60 images of each person per distance; as there are eight distances, the total number of images per person is 480 plus the identity image. Therefore, the total number of images in the dataset is 9620. The identity face is a close image taken from a smartphone with the volunteer facing the camera. The height of the identity face in pixels is approximately 800 px.

The dataset has been divided arbitrarily into two subsets of the same size. One is going to be used for the calculation of the proposed thresholds in Section 4 and the other for the comparison between the accuracy using the proposed and the original thresholds in Section 6. A summary of the dataset can be seen in Table 2.

It is worth noting that GDPR (General Data Protection Regulation) compliance is a critical consideration in our data-gathering process, as we are committed to respecting individuals’ privacy and adhering to the regulations. All the volunteers were asked to give informed consent to allow us to record their faces and to use them for the purpose of this research only.

The minimum distance of our videos is 2 m. This is a safe distance for the user with a small drone like the one used to record. At further distances, the volunteers felt more comfortable as they did not have a drone close to them. Therefore, to enhance the user experience, it is important to increase the accuracy of the face verification algorithms at long distances.

Moreover, Table 3 shows the relation between the distance of the face from the UAV and the average size in pixels of the face recorded. The large difference in size depending on the distance can be observed. These face sizes have been acquired using a 4k camera. If a lower resolution camera is used, the size of the face will be smaller too at each distance as the size of the whole image is also smaller.

**Figure 4:**
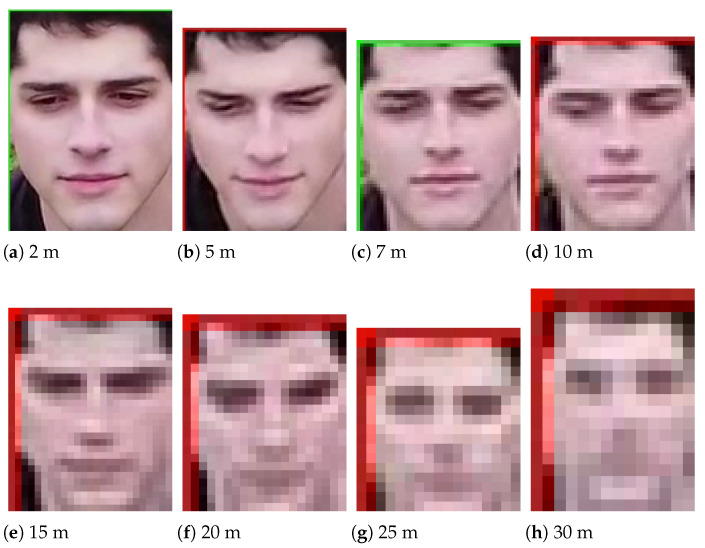
Cropped faces at the eight distances recorded.

Furthermore, Figure 4 shows the cropped faces from the eight different distances of the dataset. In Figure 4a, all features of the face can be seen without any problem. The person can easily be verified. On the other hand, in Figure 4h it is challenging to verify the person of the image due to the low resolution and its small size.

### 4.2. Empirical Definition of Face Scale Based on Distance

To be able to calculate the new dynamic thresholds, a new scale for them has to be established. Two different approaches can be adopted to define the new scale. The first one uses the distances from the face to the UAV’s onboard camera. Depending on the distance of the person, one threshold or another is applied. This approach has several problems. For example, it is hard to know exactly the distance from the face. The UAV should have a rangefinder mounted to be able to find the distance. However, not all UAVs have one mounted, thereby being unable to take this approach. Moreover, if the resolution of the camera varies, the size of the detected face in pixels will vary too. Therefore, we can have different face sizes for one distance, depending on the resolution of the camera. That is why using the distance as a scale is not efficient or useful.

The alternative and more advantageous approach is to directly utilise the size of the face for the scale. One threshold would be applied depending on the pixels of the face. Moreover, by taking this approach and knowing the resolution of the camera, the distance to the face could be calculated approximately if needed.

**Figure 5:**
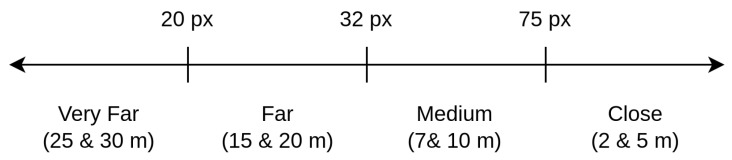
Scale of defined distances depending on the width of the cropped face.

Once the approach is determined, the scale has to be created. As Table 3 shows, there are major changes in the width rather than the height of the image. Hence, the thresholds will be decided depending on the width of the face. Then, the scale is defined using four different ranges, as Figure 5 shows. It also shows which distances of our dataset each of the ranges cover. The principle of the proposed scale scheme is simple and intuitive, whilst the performance is effective. If the detected face width is less than 20 px (pixels), it will be considered that the face is very far and the corresponding threshold will be used. If the face width is between 20 and 32 px, the person is considered to be far and a second threshold will be selected instead. If it is between 32 and 75 px, it is a medium distance, and if the face is larger than 75 px, the person is very close and then a corresponding threshold will be applied. The regions have been defined based on empirical experimentation and because those pixel values correspond to the different distances shown in Figure 5.

By using this scale and changing the thresholds depending on the detected face width, it is expected to achieve an improvement in the accuracy of the face verification algorithms at all distances. Moreover, by adopting this approach we have made the system independent of the camera resolution because it only depends on the size in pixels of the face, not the distance to the person.

### 4.3. Methodology for Empirical Threshold Calculation

Algorithm 1 shows the proposed algorithm to calculate the dynamic-distance-based thresholds for each distance, metric, and face verification algorithm.

The verification set is composed of a close image of the face of each person appearing in the dataset named as ‘face identity’. Then, the positive pairs are the frames from the dataset associated with a specific identity and the same ‘face identity’ of that person. In contrast, the negative pairs include the same ‘face identity’, along with all the remaining frames that are not associated. The similarity index is defined as the distance between two faces. Therefore, each negative or positive pair will have an associated similarity index. A hyperparameter is used to iterate along the threshold candidates in the loop. Its value depends on the metric or the face verification algorithm used.

In the algorithm, first, two ratios are calculated, one for positive pairs and another for negative ones. As the number of samples in both pairs are different, this study establishes this ratio to provide the same importance to both pairs. Otherwise, the pairs with more samples (in this case the negative pairs) will have more importance when calculating the thresholds. Afterwards, a for loop is defined from the minimum ($E_{min}^{+}$) to the maximum similarity index ($E_{max}^{+}$) of all positive pairs using as step the hyperparameter.
**Algorithm 1** Proposed optimal threshold calculation algorithm.Let us define the verification set (*V*) as one sample ($V_{i}$) of each one of the y face identifies to be verified ($V_{i}$) and a dataset (*D*), composed of x samples ($S_{j}$) of the same face identities ($F_{i}$). $V=\sum_{i=0}^{y}V\left(i\right),\;D=\sum_{i=0}^{y}\sum_{j=0}^{x}F_{i}\left(S_{j}\right)$ Let us create now the **Positive Pairs of identity** *I*, defined as: $P_{I}^{+}=\left(V_{i},\;\sum_{j=0}^{x}F_{I}\left(S_{j}\right)\right)$ And the **Negative Pairs of identity** *I*, defined as: $P_{I}^{-}=\left(V_{i},\;\sum_{i=0}^{y}\sum_{j=0}^{x}\;if\left(i\neq I\right)\left[F_{I}\left(S_{j}\right)\right],\;otherwise\;0\right)$ Let us define the **similarity index of a pair** as: E(x,y). And let us define the **minimum similarity index of all the positive pairs** as: $E_{min}^{+}=min\left(\sum_{i=0}^{y}E\left(P_{i}^{+}\right)\right)$ Analogously, let us define the **maximum similarity index of all the positive pairs** as: $E_{max}^{+}=max\left(\sum_{i=0}^{y}E\left(P_{i}^{+}\right)\right)$ Let us define the **hyperparameter** as the step used to iterate the threshold candidates in the *for* loop. The value depends on the algorithm and the metric (the cosine or Euclidean distance) used. And let us apply the proposed dynamic threshold calculation as follows: **Input:** $P_{I}^{+},\;P_{I}^{-},\;E_{min}^{+},\;E_{max}^{+},\;hyperparameter$ **Output:** Threshold,MaxAccuracy Initialize threshold=0,MaxAccuracy=0 Calculate Ratio of all Positive Pairs: $R\left(P^{+}\right)=\frac{\sum_{i=0}^{y}Size\left(P_{i}^{+}\right)}{\sum_{i=0}^{y}Size\left(P_{i}^{+}\right)+\sum_{i=0}^{y}Size\left(P_{i}^{-}\right)}$ Calculate Ratio of all Negative Pairs: $R\left(P^{-}\right)=\frac{\sum_{i=0}^{y}Size\left(P_{i}^{-}\right)}{\sum_{i=0}^{y}Size\left(P_{i}^{+}\right)+\sum_{i=0}^{y}Size\left(P_{i}^{-}\right)}$ **for** $candidate=E_{min}^{+};candidate<E_{max}^{+};candidate=candidate+hyperparameter$ 
**do**    $TP\left(TruePositives\right)=count(\sum_{i=0}^{y}if\left(E\left(P_{i}^{+}\right)<candidate\right))\;1,\;otherwise\;0$    $TN\left(TrueNegatives\right)=count(\sum_{i=0}^{y}if\left(E\left(P_{i}^{-}\right)<candidate\right))\;1,\;otherwise\;0$    *A* = 100 × $\frac{TP\times R\left(P^{+}\right)+TN\times R\left(P^{-}\right)}{\sum_{i=0}^{y}Size\left(P_{i}^{+}\right)\times R\left(P^{+}\right)+\sum_{i=0}^{y}Size\left(P_{i}^{-}\right)\times R\left(P^{-}\right)}$    **if** A>MaxAccuracy **then**        MaxAccuracy=A        Threshold=candidate    **end if** **end for**


The threshold candidate value is the one to be iterated. In the loop, the numbers of True Positives (TP) and True Negatives (TN) are calculated first. TP is calculated as the number of positive pairs whose similarity index is less than the threshold candidate. TN are the result of the number of negative pairs whose similarity index is less than the threshold candidate. Then, the accuracy is calculated using TP, TN, and the ratios previously calculated. The formula for the accuracy is as follows:

$$Accuracy=\frac{TP+TN}{TP+TN+FP+FN}$$

where FP means False Positives (negative pairs similarity indexes are higher than the threshold candidate) and FN means False Negatives (positive pairs similarity indexes are higher than the threshold candidate). The sum of TP and FN corresponds to all the positive pairs, and the sum of TN and FP corresponds to all the negative pairs. Therefore, the accuracy is calculated by dividing the sum of TP and TN by the sum of all positive and negative pairs, applying the ratios calculated.

Finally, if the accuracy is the maximum value calculated so far, the threshold candidate is the chosen threshold for this moment. When the loop finishes, the threshold candidate that achieves the highest accuracy will be the chosen one.

### 4.4. Application of the Proposed Methodology for Close (5 m) and Far (15 m) Distances

The thresholds will be calculated in order to maximise the accuracy in each range of the scale, as seen in the proposed algorithm (Algorithm 1). This section further explains how the algorithm works to maximise the accuracy.

**Figure 6:**
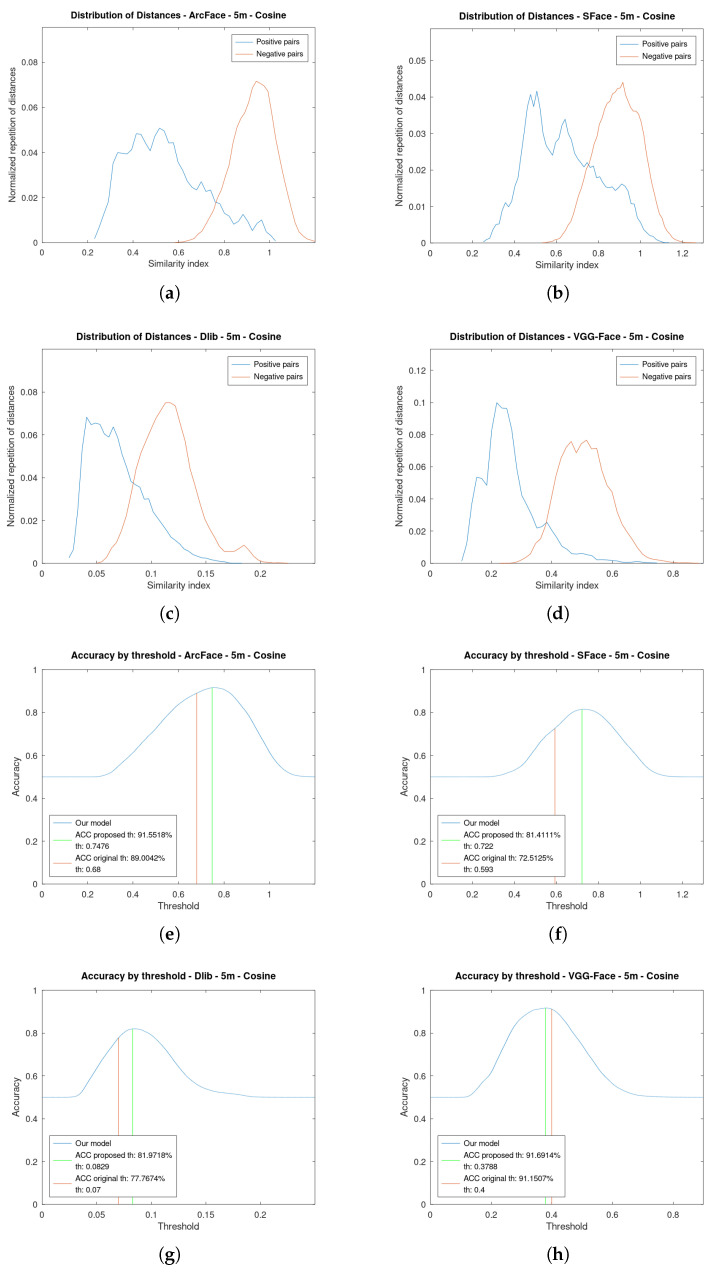
Distribution of the similarity indexes and accuracy by threshold at **5 m** of the four algorithms using **cosine distance** as metric. (**a**) Distribution of Similarity indexes of ArcFace. (**b**) Distribution of Similarity indexes of SFace. (**c**) Distribution of Similarity indexes of Dlib. (**d**) Distribution of Similarity indexes of VGG-Face. (**e**) Accuracy by threshold of ArcFace. (**f**) Accuracy by threshold of SFace. (**g**) Accuracy by threshold of Dlib. (**h**) Accuracy by threshold of VGG-Face.

**Figure 7:**
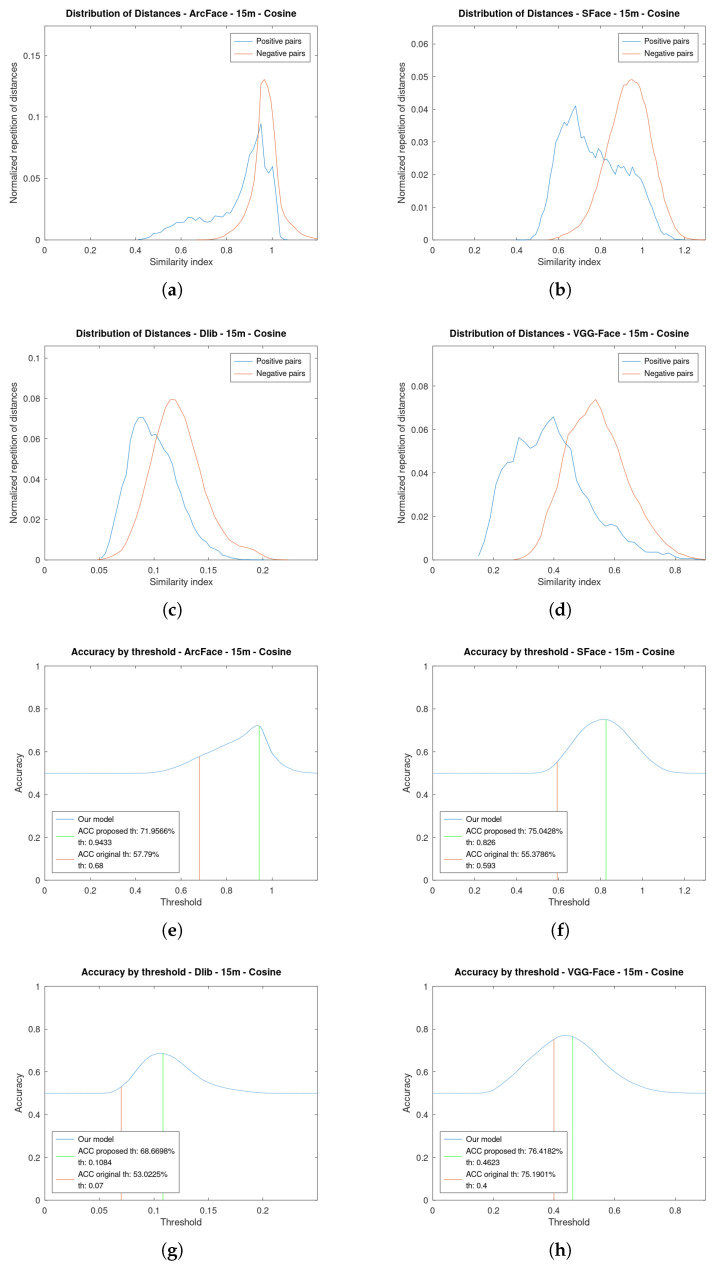
Distribution of similarity indexes and accuracy by threshold at **15 m** of the four algorithms using **cosine distance** as metric. (**a**) Distribution of Similarity indexes of ArcFace. (**b**) Distribution of Similarity indexes of SFace. (**c**) Distribution of Similarity indexes of Dlib. (**d**) Distribution of Similarity indexes of VGG-Face. (**e**) Accuracy by threshold of ArcFace. (**f**) Accuracy by threshold of SFace. (**g**) Accuracy by threshold of Dlib. (**h**) Accuracy by threshold of VGG-Face.

Figure 6 shows the graphics for the four face verification algorithms while using a cosine distance at 5 m. Figure 7 is an analogous figure for 15 m. In each of these figures, two graphs are shown per the algorithms analysed. The first graph shows the distribution of the similarity indexes. It is composed of two plots: one for the positive pairs and another one for the negative pairs. The second graph shows the value of the accuracy as a function of the threshold. Additionally, this second graph shows the two plots depicting the original threshold and the proposed one.

Regarding the distributions of the similarity indexes, they have been obtained by executing the face verification pipeline on the created dataset using the four state-of-the-art algorithms analysed: ArcFace, SFace, Dlib, and VGG-Face. By using our own dataset, we have obtained well-distributed distances both for positive and negative pairs. The positive pair distances have been obtained by executing the pipeline using the videos of one person and comparing them with their photo. The negative pairs have been obtained in the opposite way, using the videos of one person and comparing them with the photos of the other people from the dataset, as explained in the proposed algorithm. The further apart the two different plots are, the greater the accuracy that will be achieved by returning more true positives whilst minimising the false ones. If they are too overlapped, it will be difficult to verify a person and many mistakes will occur. Nevertheless, by choosing an adequate threshold the accuracy can be maximised.

Let us focus, for instance, on ArcFace at 5 m using cosine distance Figure 6a,e. In Figure 6a, it can be seen that both plots are well separated, and thus high accuracy should be achieved. The positive pairs have lower values than the negative pairs. This is because the positive pairs are more similar than the negative ones, as they should be. In Figure 6e, the accuracy across the thresholds can be seen. Below 0.2, the accuracy is 50% because all the pairs would be identified as false due to none of the similarity indexes being below the threshold. Then, we have our proposed threshold in green and the original one in orange. As shown, there is an improvement in the accuracy of choosing the proposed one.

It is worth noting that the thresholds chosen do not match the maximum of the accuracy plots. This is because what has been maximised is a range of our scale and not every distance of the UAV from the face. Therefore, what is optimised is a range of distances rather than every fixed distance.

Notice how at 15 m, for instance, Figure 7a, the plots of the distribution of the positive and negative pairs are more overlapped. This is due to the fact that the faces are increasingly different as their resolution is lowered. Therefore, it is more difficult to choose an optimal threshold. However, it can be seen in the distribution of positive pairs that there is a tail to the left, which facilitates the presence of a substantial number of true positives, even in cases of plot overlap.

All of the algorithms approximately maintain their distribution, but the positive pairs have moved to the right. This supports the necessity of varying the thresholds depending on the distance to the face.

**Figure 8:**
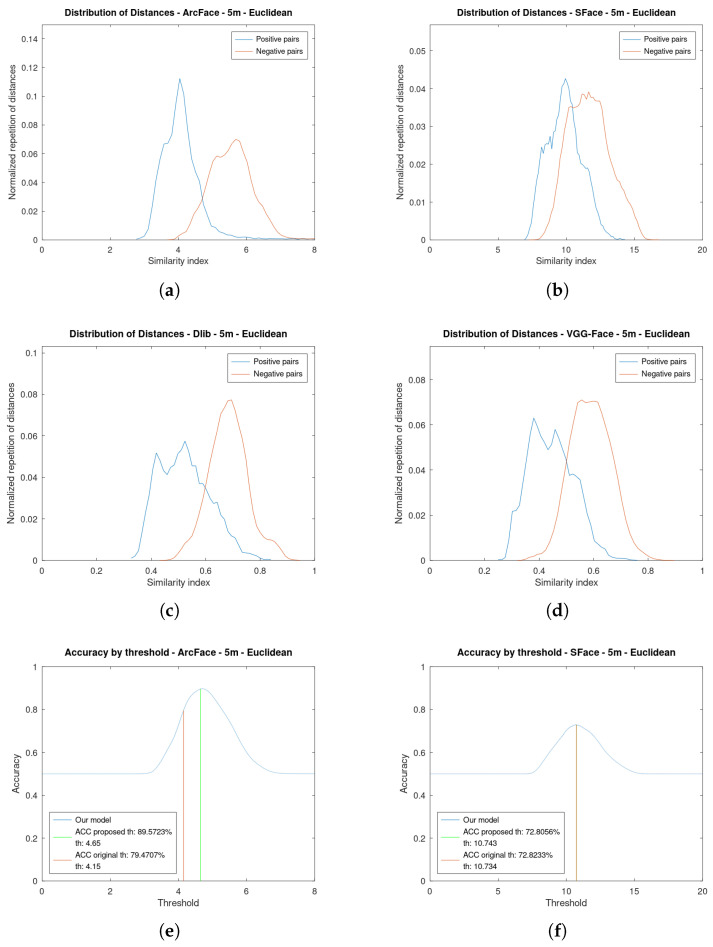

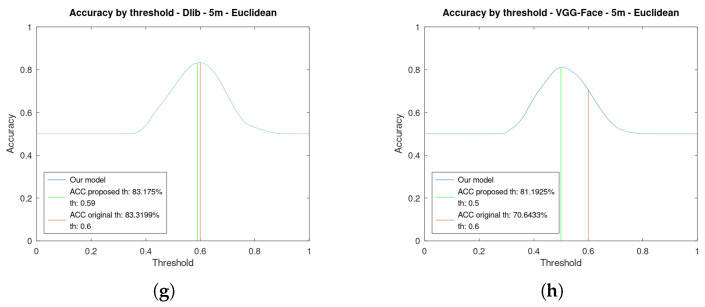
Distribution of similarity indexes and accuracy by threshold at **5 m** of the four algorithms using **Euclidean distance** as metric. (**a**) Distribution of Similarity indexes of ArcFace. (**b**) Distribution of Similarity indexes of SFace. (**c**) Distribution of Similarity indexes of Dlib. (**d**) Distribution of Similarity indexes of VGG-Face. (**e**) Accuracy by threshold of ArcFace. (**f**) Accuracy by threshold of SFace. (**g**) Accuracy by threshold of Dlib. (**h**) Accuracy by threshold of VGG-Face.

**Figure 9:**
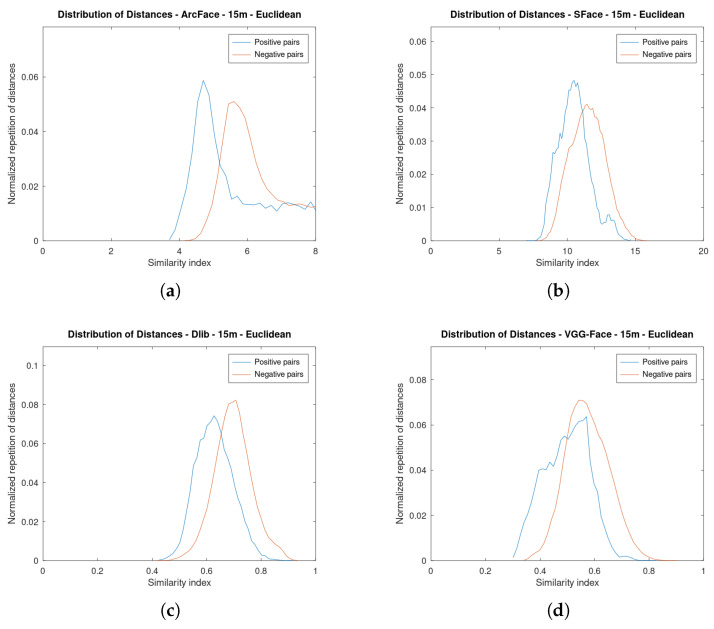

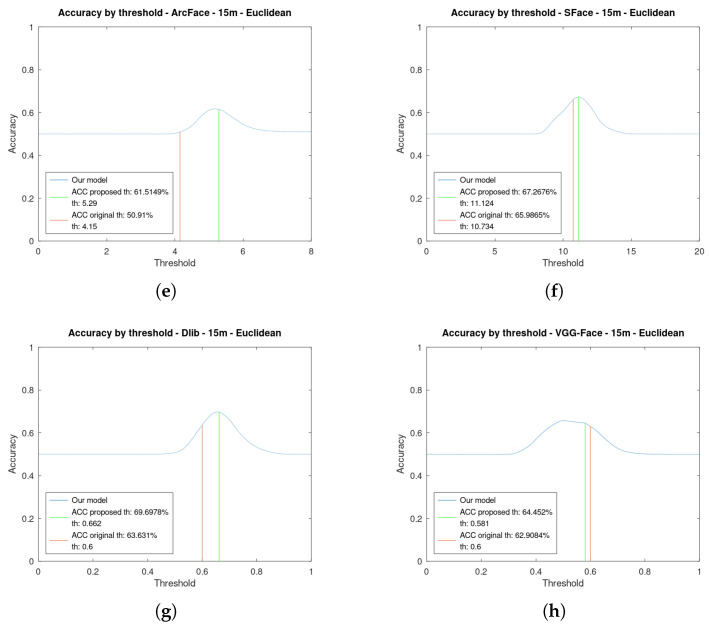
Distribution of the similarity indexes and accuracy by threshold at **15 m** of the four algorithms using **Euclidean distance** as metric. (**a**) Distribution of Similarity indexes of ArcFace. (**b**) Distribution of Similarity indexes of SFace. (**c**) Distribution of Similarity indexes of Dlib. (**d**) Distribution of Similarity indexes of VGG-Face. (**e**) Accuracy by threshold of ArcFace. (**f**) Accuracy by threshold of SFace. (**g**) Accuracy by threshold of Dlib. (**h**) Accuracy by threshold of VGG-Face.

Figure 8 and Figure 9 show analogous results but using Euclidean distance as metric. As can be seen, the plots are further apart and do not overlap much. By using Euclidean distance, the value of the thresholds is greater than in cosine because the similarity indexes also have greater values. The cosine distance range is limited between 0 and 2, while the Euclidean distance range is unlimited.

In all figures at 15 m (Figure a–d), it can be seen that the positive pairs plots are shifted to the right: the two pairs of faces are less alike. This is due to the fact that the resolution is lower as the face is further from the drone, so the two faces are going to be more different.

The use of our dynamic thresholds will not significantly increase the computational requirements of a face verification system. Instead of having one fixed threshold for all the distances, in the proposed scheme a threshold will be dynamically selected based on the distance to the person. Our technique will only add 1 us to the execution time of the pipeline. As the selection is not complex, the computational requirements or the power consumption will not vary greatly. Furthermore, our system will still work in different operational conditions. For instance, in crowded environments, the threshold will be adjusted for the size of each of the faces detected. Moreover, in adverse weather conditions, our proposed technique will also work as far as the face detection algorithm is able to detect faces correctly under such circumstances. Finally, it is worth highlighting that the empirical work of this paper is based on real implementation of the proposed system.

### 4.5. Recommended Thresholds for the Selected Face Verification Algorithms

Table 4 and Table 5 show the proposed dynamic-distance-based thresholds calculated for each range of the scale for both cosine and Euclidean distance, respectively. They are the result of applying the proposed algorithm (Algorithm 1) to every range, face verification algorithm, and metric selected.

## 5. Implementation

As mentioned before, four different face verification algorithms are explored: ArcFace, SFace, Dlib, and VGG-Face. For the empirical calculation of the threshold, a common framework where all of them are integrated is created in the same machine in this research. The implemented framework is referred to as DeepFace and includes four different face verification algorithms with their pre-trained weights and two different distance calculation metrics [35,36].

It also implements several face detection algorithms such as RetinaFace [34], MTCNN [37], Dlib [8], or Face-SSD [38]. As mentioned, RetinaFace is the face detection algorithm used in this research. It has been chosen due to its great accuracy at all distances in our dataset. It was able to detect faces even at 30 m with a small reduced number of false positives. As this paper is focused on the face verification algorithms, there is no analysis of the face detection algorithms, and, therefore, the one with the best performance on our dataset was the one chosen.

Deepface is run on Python version 3.8. The library is mainly powered by TensorFlow [39] and Keras [33]. For the preprocessing and postprocessing of the videos, OpenCV is used. The framework has been executed on the same machine using a Focal Ubuntu version 20.04.3. Pycharm is the IDE (Integrated Development Environment) used to write and execute the code. The threshold calculations were performed using GNU Octave.

The proposed pipeline has been implemented using the mentioned framework. The proposed algorithm and associated techniques have been integrated to the four state-of-the-art face verification algorithms on the same testbed for evaluation and comparison purposes.

## 6. Experimental Results

### 6.1. Testbed Description

All experiments were carried out on a single NVIDIA GeForce GTX TITAN X (Nvidia Corporation, Santa Clara, CA, USA) with 12 GB of onboard memory. The experiments were repeated five times, and the average of the results of the five runs was then calculated to obtain the final results. The UAV used to obtain the videos is a Mini 2 model created by DJI. The videos were recorded with 4K resolution (3840 × 2160 px) at 30fps.

### 6.2. Empirical Validation of the Improvement Achieved by the Dynamic Thresholding Technique Proposed for UAV-Based Face Verification

The accuracy is compared between the original thresholds and the proposed ones. Table 6 shows the accuracy using the proposed thresholds and with the cosine distance as metric, and Table 7 shows the accuracy using Euclidean distance.

**Figure 10:**
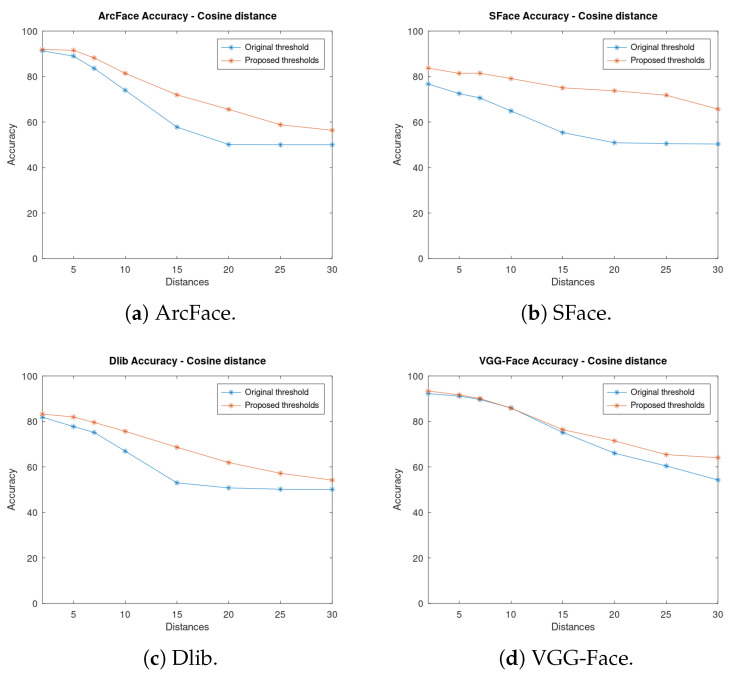
Accuracy of the algorithms at fixed distances using the original and the proposed thresholds and cosine distance as metric.

Figure 10 illustrates the accuracy of the four face verification algorithms depending on the distance using the cosine distance as the metric. It shows the accuracy both using the proposed thresholds and the original ones. It is important to mention that our proposed technique improves all the analyzed face verification algorithms, and thus it is not tailored for a concrete algorithm. For instance, in VGG-Face (Figure 10d) the best improvement is achieved at long distances, whilst at close distances, the accuracy is not greatly increased as it already had good results. Both ArcFace and Dlib have a greater improvement at medium and far distances. SFace (Figure 10b) has improved the accuracy in all instances, with more than a 25% of improvement at some distances.

**Figure 11:**
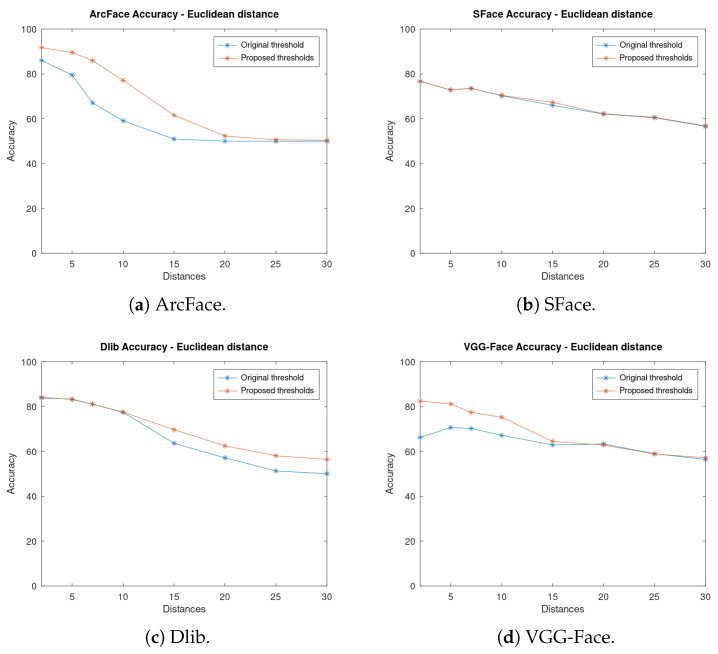
Accuracy of the algorithms at fixed distances using the original and the proposed thresholds and Euclidean distance as metrics.

Then, Figure 11 shows the accuracy of each algorithm for both thresholds using Euclidean distance as a metric. In contrast to cosine, if using the VGG-Face algorithm, a better improvement is achieved at close distances (Figure 11d), but still, the accuracy is lower than that of using cosine. In the ArcFace algorithm, similar results are obtained, and there are greater improvements at close and medium distances (Figure 11a), whilst at long distances, the accuracy does not improve much. One conclusion that can be drawn from this is that Euclidean distance is not optimal for these algorithms as the accuracy does not improve at long distances, which is where it is needed most for a UAV use case. Dlib (Figure 11c) improves at far distances, while at close distances the same results are obtained.

SFace (Figure 11b) has minimum improvement while using Euclidean distance as a metric. It can be seen that SFace does not have good accuracy while using this metric. The proposed thresholds are all very close to the original one; therefore, the improvement is minimal. This algorithm could not be improved using our method of Euclidean distance.

On the other hand, as seen previously, while using cosine distance (Figure 10b) there is a high improvement in accuracy. Therefore, it can be concluded that SFace performs far better while using cosine distance than while using Euclidean distance.

As can be seen, there is a huge improvement in accuracy when the new thresholds are used. This improvement is more significant at long distances where the accuracy was very low with the original thresholds as they were optimised for close distances. For instance, at 20 m with ArcFace and cosine distance, an improvement of 15% in accuracy has been achieved.

To the best of our knowledge, existing face verification studies simply apply a static fixed threshold to the distance between the embeddings as their thresholding technique. In these results, we have shown that when executing face verification algorithms from drones, the accuracy can be significantly improved by applying a dynamic threshold depending on the distance instead of using a fixed threshold.

Our system has some limitations that are the same as the ones of the face verification algorithms being used. If the algorithm cannot perform correctly at a specific distance, our dynamic thresholds will not be able to increase its accuracy significantly.

### 6.3. Empirical Validation of the Best Similarity Index Based on the Selected Algorithm

Table 8 shows, with each metric, at which distances the greatest improvements are achieved. Also, the final column shows which metric is better to use with each algorithm. Dlib is the only one that achieves a greater performance using Euclidean distance at all distances.

It is worth mentioning that the proposed technique is able to enhance the accuracy of all the proposed face verification algorithms using any of the similarity metrics analysed. Thus, it has been proven that the methodology and approach provided in this contribution is robust enough to be considered in this kind of AI pipeline.

### 6.4. Analysis of Inference Times

**Figure 12:**
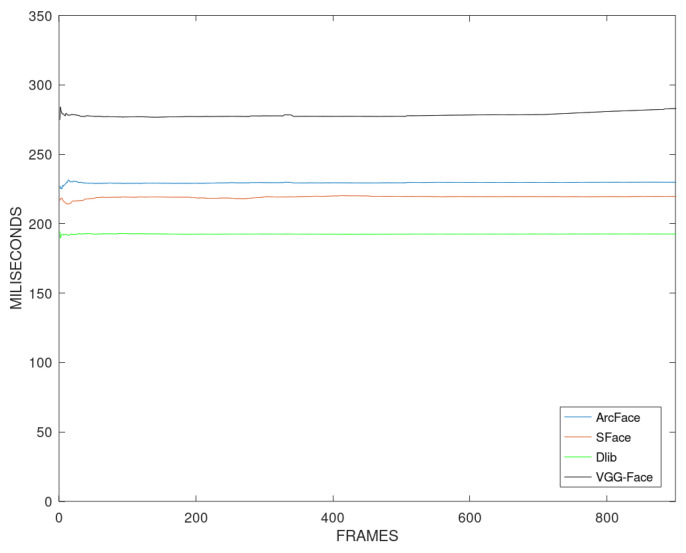
Cumulative average of the inference time in milliseconds per frame for each algorithm.

The speed of the pipeline is assessed by measuring the inference time. It is defined as the time spent since the frame enters the processing pipeline until the decision is received, and it is measured empirically to compare the speed of the algorithms. Figure 12 shows the cumulative average inference time per frame. A great difference between the face verification algorithms can be appreciated.

VGG-Face is by far the slowest algorithm. When used, the inference time takes approximately 275 ms, and we can only achieve a maximum processing speed of 4 fps. On the other hand, Dlib is the fastest algorithm. The pipeline processes each frame in approximately 180 ms. Furthermore, it is noted that RetinaFace takes approximately 165 ms, which means that Dlib can obtain the features in less than 15 ms.

The second fastest algorithm is SFace, with approximately 220 ms per frame. Finally, ArcFace is the second slowest algorithm, taking approximately 230 ms per frame to obtain the result of the pipeline.

Our distance-based thresholds do not add any significant delay to the system as one threshold or another will be selected based on the size of the face. Our technique will only add 1 us to the execution time of the pipeline. The calculation of the thresholds using the proposed algorithm will be made before the system is deployed. Therefore, the threshold selection will be almost instant, and real-time processing could be achieved by improving the face detection and verification algorithms.

## 7. Concluding Remarks

This paper presents a novel technique to perform face verification by modifying the thresholds depending on the distance of the UAV from the face. Moreover, an empirical study of four state-of-the-art face verification algorithms has been performed including a comparison with the proposed technique.

By adding the dynamic size-adaptive thresholds to the face verification pipeline, we have significantly improved the accuracy of these face verification algorithms. Furthermore, two metrics have been employed to conduct the comparison (cosine and Euclidean distance). Another analysis has been performed to conclude which of the metrics better suits each algorithm in order to achieve higher accuracy for face verification.

Empirical results have shown that a high improvement in the accuracy of the face verification algorithms at different distances has been achieved. Using the ArcFace algorithm at 20 m with cosine distance as a metric, there is an improvement of 15% in the accuracy. The SFace algorithm has been improved by more than 20% at some distances. At 30 m and using cosine distance, the VGG-Face algorithm has improved its accuracy by 10%.

Our proposed thresholding algorithm has improved all four face verification algorithms used. This proves that by using a dynamic threshold, the accuracy of the face verification algorithms can be improved, leading to fewer false positives and maximising the true ones. Furthermore, our technique is not limited to UAV-based public safety use cases and can be applied to any application domain where face verification is required, such as intruder detection in industrial premises.

For future work, an enhanced dataset can be created using more people at more distances and angles from the UAV to the face. The lighting is important too because in the dataset for this research, the data were obtained in various light conditions. For future research, more lighting conditions can be considered to have a more complex and complete dataset, for example, at night or low visibility.

Moreover, the thresholds have been calculated to maximise the accuracy, but other metrics can be maximised. For example, the same calculations can be made but maximising the F1-Score or defining a maximum acceptable value of false positives. The maximised metric would vary depending on the use case.

Some potential future research directions involve integrating other techniques to improve the accuracy of the algorithms depending on the environment. For instance, light or image enhancement methods can be added to increase the brightness or resolution of the images. Furthermore, the backbones of the algorithms can also be modified to increase the accuracy by adding or modifying layers while trying not to reduce their speed.

## Figures and Tables

**Table 2:** Specifications of the novel created dataset.

**Number of people**	20 volunteers
**Distances recorded**	2, 5, 7, 10, 15, 20, 25 and 30 m
**Videos resolution**	4K (3840 × 2160 px)
**Videos frame rate**	30 FPS
**Recording device**	DJI Mini 2 Drone
**Duration of each video**	30 s
**Total number of images**	9620
**Age range**	18–60 years
	Different ethnicities
**Other characteristics**	Different genders
	Different lighting conditions
**Dataset division**	50% Tresholds Calculation
	50% Testing

**Table 3:** Distance from the camera to the face versus the size of the detected face in pixels.

Distance	Size Face
2 m	125 × 170 px
5 m	80 × 98 px
7 m	50 × 59 px
10 m	38 × 44 px
15 m	25 × 31 px
20 m	20 × 24 px
25 m	17 × 19 px
30 m	14 × 19 px

**Table 4:** Calculated dynamic thresholds for cosine distance as metric.

Algorithm	Very Far	Far	Medium	Close
ArcFace	0.9522	0.9433	0.7958	0.7476
SFace	0.9	0.826	0.772	0.722
Dlib	0.1245	0.1084	0.0882	0.0829
VGGFace	0.5241	0.4623	0.3924	0.3788

**Table 5:** Calculated dynamic thresholds for Euclidean distance as metric.

Algorithm	Very Far	Far	Medium	Close
ArcFace	9.65	5.29	4.91	4.65
SFace	10.916	11.124	10.659	10.743
Dlib	0.721	0.662	0.603	0.59
VGGFace	0.62	0.581	0.528	0.5

**Table 6:** Accuracy of the algorithms at different distances using our dynamic thresholds—Cosine Distance.

Algorithm	2 m	5 m	7 m	10 m	15 m	20 m	25 m	30 m
**ArcFace**	91.91	91.55	88.19	81.41	71.96	65.59	58.82	56.39
**SFace**	83.74	81.41	81.48	79.13	75.04	73.76	71.81	65.70
**Dlib**	83.26	81.97	79.58	75.68	68.67	61.95	57.21	54.22
**VGG-Face**	93.39	91.69	90.09	85.87	76.42	71.49	65.43	64.10

**Table 7:** Accuracy of the algorithms at different distances using our dynamic thresholds—Euclidean distance.

Algorithm	2 m	5 m	7 m	10 m	15 m	20 m	25 m	30 m
**ArcFace**	91.69	89.57	85.91	77.17	61.52	52.28	50.58	50.41
**SFace**	76.70	72.81	73.51	70.41	67.27	62.23	60.64	56.80
**Dlib**	84.19	83.18	81.07	77.55	69.70	62.47	58.07	56.42
**VGG-Face**	82.47	81.19	77.36	75.28	64.45	62.83	58.84	57.17

**Table 8:** Distances at which the greatest improvements are achieved with each metric and what is the recommended metric to use with each algorithm.

Algorithm	Improvement with Cosine Distance	Improvement with Euclidean Distance	Metric Recommended
**ArcFace**	All distances	Close Distances	**Cosine Distance**
**SFace**	All distances	None distance	**Cosine Distance**
**Dlib**	All distances	Far distances	**Euclidean Distance**
**VGG-Face**	Far distances	Close distances	**Cosine Distance—Close Distances Euclidean Distance—Far Distances**

## Data Availability

The novel UAV-recorded dataset used in the current study is not publicly available due to the General Data Protection Regulation (GDPR) requirements as it contains personally identifiable information.
